# *Clostridium difficile* Infection Seasonality: Patterns across Hemispheres and Continents – A Systematic Review

**DOI:** 10.1371/journal.pone.0120730

**Published:** 2015-03-16

**Authors:** Luis Furuya-Kanamori, Samantha J. McKenzie, Laith Yakob, Justin Clark, David L. Paterson, Thomas V. Riley, Archie C. Clements

**Affiliations:** 1 Research School of Population Health, The Australian National University, Canberra, Australian Capital Territory, Australia; 2 School of Population Health, The University of Queensland, Herston, Queensland, Australia; 3 London School of Hygiene and Tropical Medicine, Department of Disease Control, London, United Kingdom; 4 Drug ARM Australasia, Annerley, Queensland, Australia; 5 The University of Queensland, UQ Centre for Clinical Research, Herston, Queensland, Australia; 6 Microbiology & Immunology, The University of Western Australia and Department of Microbiology PathWest Laboratory Medicine, Queen Elizabeth II Medical Centre, Nedlands, Western Australia, Australia; Cleveland Clinic, UNITED STATES

## Abstract

**Background:**

Studies have demonstrated seasonal variability in rates of *Clostridium difficile* infection (CDI). Synthesising all available information on seasonality is a necessary step in identifying large-scale epidemiological patterns and elucidating underlying causes.

**Methods:**

Three medical and life sciences publication databases were searched from inception to October 2014 for longitudinal epidemiological studies written in English, Spanish or Portuguese that reported the incidence of CDI. The monthly frequency of CDI were extracted, standardized and weighted according to the number of follow-up months. Cross correlation coefficients (XCORR) were calculated to examine the correlation and lag between the year-month frequencies of reported CDI across hemispheres and continents.

**Results:**

The search identified 13, 5 and 2 studies from North America, Europe, and Oceania, respectively that met the inclusion criteria. CDI had a similar seasonal pattern in the Northern and Southern Hemisphere characterized by a peak in spring and lower frequencies of CDI in summer/autumn with a lag of 8 months (XCORR = 0.60) between hemispheres. There was no difference between the seasonal patterns across European and North American countries.

**Conclusion:**

CDI demonstrates a distinct seasonal pattern that is consistent across North America, Europe and Oceania. Further studies are required to identify the driving factors of the observed seasonality.

## Introduction

*Clostridium difficile* is the most common cause of antibiotic-associated diarrhea among hospital inpatients [1]. The incidence and severity of *C*. *difficile* infection (CDI) have increased worldwide in the last two decades [2].

Understanding the seasonal patterns of infectious diseases is crucial to identify factors associated with an increased risk of infection and to implement control measures during the time of year when interventions are likely to have the greatest impact [3]. Epidemiological studies have documented a seasonal variation in the frequency of CDI, yet the mechanisms responsible for its variability remain poorly understood. Specifically, in the USA and Canada, the incidence of CDI has been reported to increase during boreal winter months (February–March) [4–6]. Antibiotic exposure is strongly associated with CDI [7–10]; consequently, it has been proposed that the observed CDI seasonality in the Northern Hemisphere is associated with the higher incidence of respiratory infections, which leads to an increase in antibiotic prescriptions during winter months [11,12].

In Australia, even though antibiotic consumption also peaks during winter (August) [13]; recent epidemiological studies have found that the seasonal pattern of *C*. *difficile* is not characterized by an increased number of CDI during winter months [14,15]. This indicates that CDI in Australia may not conform to currently proposed mechanisms of *C*. *difficile* seasonality, suggesting that factors in addition to antibiotic exposure might be driving the seasonality. Therefore, the aim of the current review was to pool the existing evidence to describe the global patterns of CDI seasonality and to facilitate improved understanding of underlying mechanisms.

## Methods

The Preferred Reporting Items for Systematic Reviews and Meta-Analyses (PRISMA) guidelines were followed in this systematic review [16]. A systematic search was undertaken in three medical and life sciences databases (PubMed, Embase and Latin American and Caribbean Health Sciences Literature [LILACS]) from their inception to October 1^st^ 2014 for longitudinal epidemiological studies that reported the incidence of CDI. Search terms included were “*Clostridium difficile*” and “season”, the specific keywords and connectors used in the systematic search strategy for each database are listed in S1.A Search strategy.

The inclusion of studies was restricted to human studies, full-text articles or abstracts written in English, Spanish or Portuguese. Studies with at least 12 months follow-up that reported the incidence of CDI or the proportion of stool specimens examined in which *C*. *difficile* was detected, per month or per season, were included. CDI intervention studies were excluded from the review because of the interference that interventions might have on transmission dynamics. Exclusions were also made for studies that reported the number of positive samples detected for *C*. *difficile* without reporting the total number of samples that were tested; unless the authors stated that the number of stool samples examined per month was constant across the follow-up period. Corresponding authors were contacted for further information regarding the total number of samples examined per month/season. The characteristics of the excluded studies are listed in S1 Table.

Two authors (LFK and LY) independently examined all the citations by title and abstracts for studies that met the inclusion criteria. Full-text version articles of all potentially relevant studies were retrieved and independently extracted. Data presented in a graphical format were extracted using Plot Digitizer version 2.6.6 (http://plotdigitizer.sourceforge.net/). Data from all the included studies were extracted and summarized in a spreadsheet. The extracted data were cross-checked by the two authors, discrepancies during the selection of studies or data extraction were resolved through discussion and consensus. The quality of the selected studies was assessed independently by the same two authors using the Newcastle-Ottawa scale (NOS) [17].

The extracted data (incidence of CDI or proportion of positive stool specimens for *C*. *difficile*) were standardized to have a mean = 0, a minimum value = −1, and a maximum value = 1 for comparison across studies. A weight between zero and 1 was assigned to each study proportional to the number of months of follow-up. The number of months of follow-up for each study were divided by the number of months of follow-up of the study with the longest follow-up period; this ensured that the study with the longest follow-up period received a weight of 1.

The weighted average of the standardized monthly incidence were then plotted by hemispheres and continents to compare the seasonal patterns of CDI in each setting. An additional plot in which weighted average of the standardized CDI data from the Southern Hemisphere was shifted 6 months to align the meteorological seasons between hemispheres was created for ease of comparison.

Cross correlation coefficients (XCORR) were used to examine the correlation and lag value (in months) between the weighted average of standardized monthly incidence of CDI across hemispheres and continents using the extracted temporal data.

## Results

**Fig 1:**
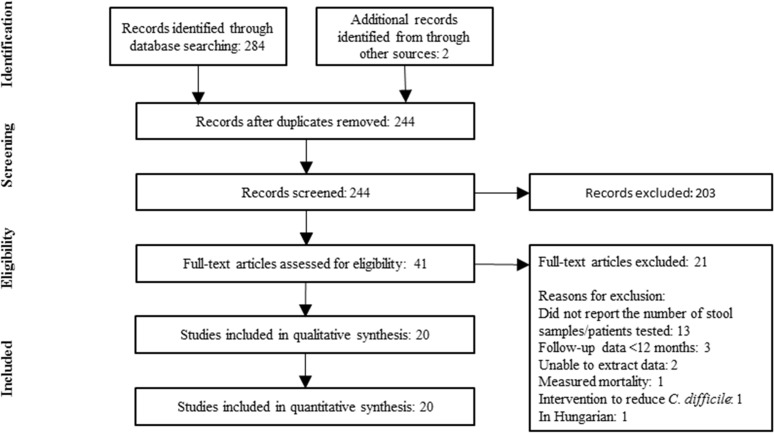
PRISMA (Preferred Reporting Items for Systematic Reviews and Meta-Analysis) flowchart of the literature search conducted on the 1^st^ October 2014.

The search identified 244 publications; after screening the publications by title and abstract, 171 publications were excluded. After a full-text review of 41 publications was conducted, 20 studies met the inclusion criteria and were selected for the review (Fig. 1). Of the 20 studies, 18 were conducted in Northern Hemisphere countries and only 2 in the Southern Hemisphere. Among the studies from the Northern Hemisphere, 13 were from North America and 5 from Europe. The 2 studies from the Southern Hemisphere were from Oceania (Australia; Tables 1 and 2). No studies from South America, Africa or Asia were identified despite additional efforts to target these regions in our search strategy (S1.B Search strategy). Using the NOS, all the studies but two were identified as high quality (⩾80% NOS score; Table 1 and S2 Table).

**Table 1:** Characteristics of included studies.

	Location	Data source	Start	Finish	Follow-up (months)	NOS scores
Archibald *et al*., 2004 [4]	All USA	National Nosocomial Infections Surveillance System	Jan 1987	Dec 2001	180	3/5
Brown *et al*., 2013 [11]	All USA	U.S. National Hospital Discharge Survey	Jan 1993	Dec 2008	192	8/9
Burckhardt *et al*., 2008 [40]	Saxony, Germany	State of Saxony Surveillance	Jan 2002	Dec 2006	60	4/5
Camacho-Ortiz *et al*., 2009 [41]	Mexico City, Mexico	Instituto Nacional de Ciencias Médicas y Nutrición Salvador Zubiran	Jan 2003	Dec 2007	60	4/5
Damani *et al*., 2011 [42]	Armagh, Northern Ireland	Craigavon Area Hospital	Jan 2007	May 2010	41	3/5
Deorari *et al*., 1999 [34]	Alberta, Canada	Alberta Children’s Hospital	Apr 1993	Mar 1995	24	9/9
Dubberke *et al*., 2009 [43]	MO, MA, OH, IL, UT (USA)	Five hospitals	Jul 2000	Jun 2006	72	5/5
Faires *et al*., 2014 [44]	Ontario, Canada	A community hospital in Southern Ontario	Aug 2006	Feb 2011	55	5/5
Furuya-Kanamori *et al*., 2014 [14]	Queensland, Australia	Sullivan Nicolaides Pathology	May 2003	Dec 2012	117	4/5
Gilca *et al*., 2010 [5]	Quebec, Canada	MED-ECHO and Quebec's provincial surveillance	Apr 1998	Mar 2006	97	4/5
Gilca *et al*., 2012 [12]	Quebec, Canada	Quebec's provincial surveillance	Jan 2005	Dec 2008	48	8/9
Jagai and Naumova, 2009 [6]	All USA	Centers for Medicare and Medicaid Services	Jan 1993	Dec 2004	144	4/5
MacDonald *et al*., 1993 [45]	Manitoba, Canada	A tertiary care referral hospital	May 1990	May 1992	23*	5/5
McFarland *et al*., 2007 [46]	Washington, USA	Veterans Administration Puget Sound Health Care System	Jan 2004	Dec 2004	12	8/9
Reil *et al*., 2012 [47]	Northern Bavaria, Germany	Synlab Medical Care Service Centre Wieden	Jan 2000	Dec 2009	120	4/5
Reveles *et al*., 2014 [48]	All USA	U.S. National Hospital Discharge Survey	Jan 2001	Dec 2010	120	4/5
Slimming *et al*., 2014 [15]	All Australia (except Northern Territory)	450 public hospitals	Jan 2011	Dec 2012	24	5/5
Sonnenberg, 2009 [49]	All England	Hospital Episode Statistics	Apr 1995	Mar 2006	132	4/5
von Muller *et al*., 2011 [50]	Saarland, Germany	The University of Saarland Hospital	Apr 2008	Jun 2010	27	4/5
Wong-McClure *et al*., 2012 [30]	NR, Costa Rica	A tertiary care hospital	Jan 2009	Jun 2011	30	4/5

*NOS*: Newcastle-Ottawa Scale, *NR*: Not reported, *MO*: Missouri, *MA*: Massachusetts, *OH*: Ohio, *IL*: Illinois, *UT*: Utah

* January 1991 not included, a nursing strike made data unretrievable.

**Table 2:** Measures of monthly *C*. *difficile* infection incidence.

	Jan	Feb	Mar	Apr	May	Jun	Jul	Aug	Sep	Oct	Nov	Dec
Archibald *et al*., 2004 [4] (Cases/10,000 patient-days)	4.25	4.46	4.69	3.21	3.93	2.53	2.28	1.97	1.01	2.60	1.19	2.15
Brown *et al*., 2013 [11] (Cases/1,000 discharges)	8.36	8.42	8.67	8.75	8.65	8.35	8.19	8.22	8.27	7.97	7.71	7.90
Burckhardt *et al*., 2008 [40] (Cases/100,000 persons)	6.15	6.15	6.67	6.67	6.67	5.60	5.60	5.60	6.28	6.28	6.28	6.15
Camacho-Ortiz *et al*., 2009 [41] (Cases/1,000 discharges)	7.97	9.63	7.08	10.55	8.85	11.06	9.92	14.37	16.44	3.97	5.06	5.46
Damani *et al*., 2011 [42] (Percentage of positive samples)	10.48	10.36	10.16	6.58	7.53	9.30	11.17	13.43	12.93	11.96	15.40	12.98
Deorari *et al*., 1999 [34] (Percentage of positive samples)	22.64	14.64	9.36	19.73	0.09	33.18	41.91	35.36	44.27	49.91	42.27	25.55
Dubberke *et al*., 2009 [43] (Cases/10,000 patient-days)	9.02	9.30	9.39	10.03	9.17	9.13	8.28	9.32	9.32	9.43	9.36	9.28
Faires *et al*., 2014 [44] (Cases/10,000 patient-days)	0.18	0.79	0.90	1.66	1.59	1.50	1.45	1.18	1.49	0.70	1.50	1.11
Furuya-Kanamori *et al*., 2014 [14] (Percentage of positive samples)	13.42	13.39	12.55	10.07	11.24	13.39	13.08	13.33	12.84	14.22	14.09	14.67
Gilca *et al*., 2010 [5] (Cases/1,000 discharges)	11.29	10.10	9.08	8.29	7.83	6.92	7.31	8.02	8.63	10.05	10.43	11.50
Gilca *et al*., 2012 [12] (Cases/1,000 discharges)	12.76	11.82	11.85	11.04	10.09	8.99	8.70	7.65	7.30	7.70	8.12	8.40
Jagai and Naumova, 2009 [6] (Cases/10,000 persons)	0.53	0.48	0.49	0.51	0.50	0.46	0.50	0.50	0.49	0.54	0.47	0.54
MacDonald *et al*., 1993 [45] (Cases/100,000 patient-days)	2.97	5.49	2.39	5.49	4.33	5.92	8.97	3.98	4.96	4.93	9.50	2.94
McFarland *et al*., 2007 [46] (Cases/10,000 patient-days)	21.90	40.51	42.70	28.47	36.86	15.33	11.31	23.36	24.09	34.67	34.67	31.02
Reil *et al*., 2012 [47] (Percentage of positive samples)	10.99	10.99	10.96	10.96	10.96	11.44	11.44	11.44	10.94	10.94	10.94	10.99
Reveles *et al*., 2014 [48] (Cases/1,000 discharge)	6.60	7.00	7.60	6.70	7.30	7.00	6.80	7.00	6.70	7.10	6.00	6.90
Slimming *et al*., 2014 [15] (Cases/10,000 patient-days)	3.32	3.32	3.80	3.80	3.80	3.53	3.53	3.53	4.27	4.27	4.27	3.32
Sonnenberg, 2009 [49] (Cases/1,000 admissions)	0.80	0.92	1.03	1.03	0.92	0.76	0.68	0.60	0.69	0.58	0.69	0.66
von Muller *et al*., 2011 [50] (Percentage of positive samples)	7.35	7.06	8.42	9.12	6.51	9.26	5.11	7.91	10.34	6.36	11.29	10.74
Wong-McClure *et al*., 2012 [30] (Cases/10,000 patient-days)	10.68	15.19	6.69	12.62	7.53	8.02	8.67	4.13	7.41	5.14	9.74	8.50

**Fig 2:**
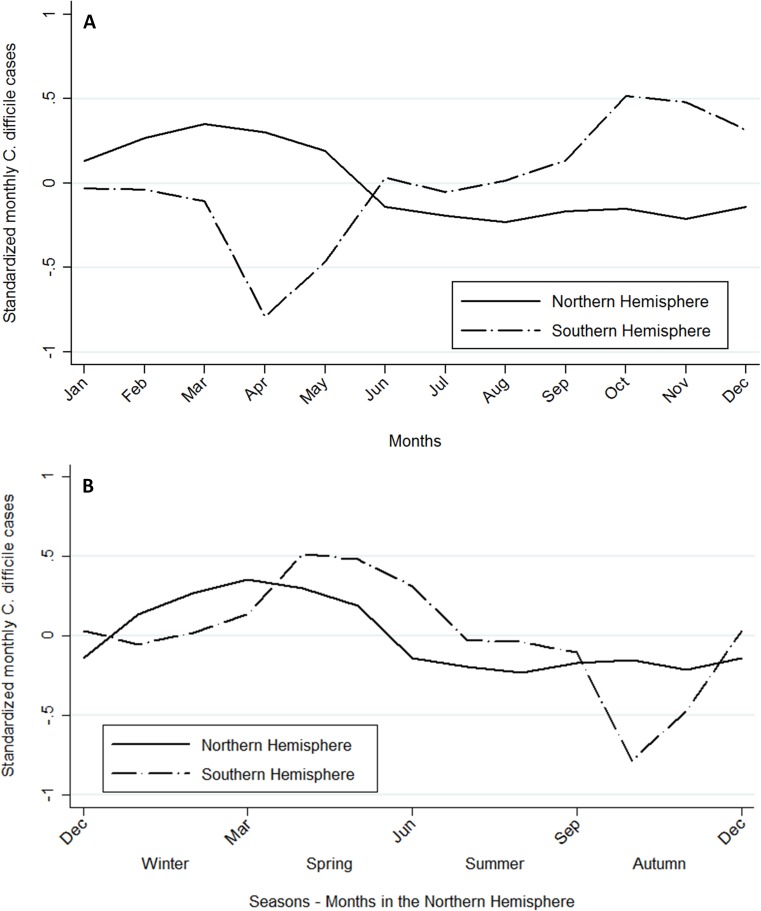
(a)Weighted average of the standardized monthly incidence of *C*. *difficile* infection by hemisphere. (b)Weighted average of the standardized monthly incidence of *C*. *difficile* infection by hemispheres. For ease of comparison, the Southern Hemisphere plot was moved 6 months (in the x-axis) thus the meteorological seasons align between hemispheres.

A similar seasonal pattern was observed between the Northern and Southern Hemisphere. In the Northern Hemisphere, CDI rates peaked during March – April (early boreal spring) and were at their lowest during the second half of the year. CDI increased in the Southern Hemisphere during the second half of the year and peaked in the last trimester of the year (October – November, the mid austral spring – Fig. 2A and 2B). The XCORR peaked (0.60) at lags = 8, indicating that the rise in the weighted average of the standardized monthly incidence of CDI in the Southern Hemisphere lagged the Northern Hemisphere by 8 months (i.e. it occurred two months later relative to the onset of spring in the Southern Hemisphere as compared to the Northern Hemisphere). The lowest value was identified (–0.76) at lag = 1, which indicates that at lag = 1 month the weighted average of the standardized monthly incidence of CDI in the Northern Hemisphere decreases while it increases in the Southern Hemisphere.

**Fig 3:**
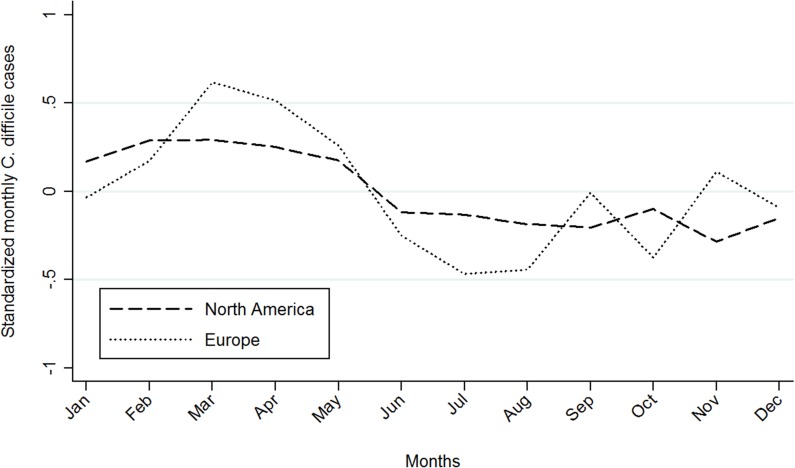
Weighted average of the standardized monthly incidence of *C*. *difficile* infection by Northern Hemisphere continents.

When the studies were grouped by continents, a similar trend was observed in the Northern Hemisphere between North American and European countries. This observation was confirmed by the peak of XCORR = 0.69 at lag 0 months. Both presented a higher frequency of CDI during the first half of the year, with peaks of CDI in March and April in Europe and North America, respectively (Fig. 3).

## Discussion

The findings of the current systematic review suggested that the Northern and Southern Hemisphere countries exhibit similar seasonal patterns characterized by CDI peaking in spring and being at their lowest during summer/autumn months. Antibiotic consumption in the community also follows a seasonal pattern. In North American and European countries the consumption of antibiotics mainly peaked in January-February, whereas in Australia antibiotic consumption peaked in August [13]. Hensgens *et al*. found that after cessation of antibiotic therapy, patients remain at higher risk of CDI for up to 3 months [18]. Therefore, the observed seasonality may indicate a lag of 2–3 months between antibiotic exposure and CDI. It is not surprising that several studies have found co-seasonality of CDI and respiratory tract infection [11,12,19]. In these studies, the respiratory infections often lead CDI by 1 month which could be explained by the corresponding incidence of respiratory tract infection and antibiotic prescription in the community [20].

Risk factors in addition to antibiotic exposure such as environmental variables (temperature, precipitation, altitude, etc.) could also be involved in the observed seasonality as they have also been demonstrate to affect the dynamics of numerous infectious diseases [3,21]. In a previous study we found that the odds of CDI infection increased by 9% (OR: 1.09; 95%CI: 1.02 to 1.17) per 100 mm increase in monthly rainfall in Queensland, Australia [14]. Respiratory tract infection transmission dynamics are highly dependent on environmental factors [21]; therefore, caution is advised for future studies drawing an association between CDI and environmental factors because of the possible confounder of co-seasonality in CDI and respiratory infections. Because CDI was traditionally viewed as a nosocomial disease, studies that assess the relationship between environmental factors and CDI are scant and this is a research gap that requires substantial development. The observed difference of two-month lag between the Southern and Northern Hemisphere (relative to the onset of spring) may be explained by the climatic zones where the studies included in the review are located. Australia, which is located in tropical and sub-tropical zones was the only country included in the review from the Southern Hemisphere; whereas the Northern Hemisphere countries included were mainly located in a temperate zone (USA, Canada, Germany, Ireland, and England). Von Boeckel *et al*. found that countries further from the equator (temperate zone) have a prominent seasonal pattern in antibiotic consumption characterized by peaks during winter, whereas antibiotic consumption is fairly constant across the months in countries located in tropical and sub-tropical zones [13]. Furthermore, Tamerius *et al*. described a similar one-month lag between the start of influenza epidemic in temperate Northern Hemisphere countries (November, end of boreal autumn) and the start of influenza epidemic in Australia (June, start of austral winter)[22]. In both cases, the influenza epidemic starts 3–4 months before the peak of CDI (March – April in Northern Hemisphere and October – November in Southern Hemisphere).

Despite contrasting antibiotic prescribing practices in outpatients between North America and Europe, the results indicate a similar seasonal pattern between European and North American countries. Patrick *et al*. found that the antibiotic consumption in the community was higher in British Columbia, Canada, than in Sweden, Germany, United Kingdom, Denmark and The Netherlands [23]. Of particular interest is the high consumption rate found in Canada compared to Denmark for some antibiotic classes such as fluoroquinolones (1.44 versus 0.15 defined daily doses [DDDs]/1000 inhabitant-days), macrolides (1.59 versus 0.92 DDDs/1000 inhabitant-days), and cephalosporins (1.86 versus 0.02 DDDs/1000 inhabitant-days) as these antibiotic classes have been associated with an increased risk of community-acquired CDI [7,8]. A similar trend in antibiotic prescribing was observed in children; higher rates of use of cephalosporins (89.1 versus 0.2 prescriptions/1000 children), lincosamides (2.3 versus 0.1 prescriptions/1000 children), macrolides (148.0 versus 42.6 prescriptions/1000 children), and fluoroquinolones (1.4 versus 0.5 prescriptions/1000 children) were reported in Canada compared to Denmark [24]. This finding supports the need to investigate additional factors (other than antibiotic exposure [11,12]) that would contribute towards a broader understanding of CDI seasonality.

Exposure to proton pump inhibitor (PPI) [25] and glucocorticoid [26] has been associated with an increased risk of CDI, however no study has yet examined the temporal relationship between monthly PPIs or glucocorticoids prescription rates and CDI seasonality. Additional factors such as the introduction of new strains of the pathogen via trade in livestock, commodities and/or movement of people (asymptomatic colonized patients such as tourists or business travellers, or hospital transfers) across boundaries should be evaluated when assessing possible factors associated with the seasonality of CDI [27]. Rodriguez-Palacios *et al*. reported a possible seasonality in contamination of retail meat in Canada with higher prevalence of *C*. *difficile* in January – February (11.5%) compared to other months of the study (4.0%) [28]. Riley has implicated the importation of onions and garlic from USA and Mexico into Australia in the increase in CDI during October – December in Western Australia [29].

Although a comprehensive review was carried out, several limitations were noted. First, only two studies were identified that reported the seasonality of CDI in Southern Hemisphere countries [14,15]. Furthermore, both studies were conducted in Australia. This may limit the generalizability of the findings for Southern Hemisphere countries only to Australia. However, the identified gap in information should encourage further investigation particularly in countries in South America, Africa and Asia. Second, there was a small number of studies from countries located between the Tropic of Cancer and the Tropic of Capricorn. The study conducted by Wong-McClure *et al*. [30] in Costa Rica was the only study from the Northern Hemisphere located in a tropical zone, precluding the comparison between the seasonality of CDI in temperate and sub-tropical/tropical climates. Despite the documented changes in CDI epidemiology [2], the increase in community-acquired CDI [31], and the different risk profiles between community- and hospital-acquired CDI patients [32], our study was also limited by the inability to compare the community- and hospital-acquired CDI seasonal patterns. Despite the increasing incidence of CDI among the paediatric population [33] only one study (Deodari *et al*. [34]) was identified that described the CDI seasonality in children; therefore, generalizability of the findings may be limited among this population. Potential factors that may contribute to differences in monthly CDI incidence that could not be accounted for in this review, such as hospital characteristics (e.g. staffing, overcrowding), CDI diagnosis ascertainment, severity of underlying illness, infection control practices, and CDI strain need to be assessed in future studies.

As the studies included in the review reported the measures of monthly CDI using different units, the values were standardized to compare the monthly CDI incidence across the studies. By doing so, the magnitude of the seasonality measured by the amplitude between the peak and the trough was lost. Although, the magnitude of the seasonality could be masked, the observed patterns should not be affected by the standardization. Finally, the weight allocated to each study was based on the number of follow-up months and not on the sample size as the number of participants or stool samples examined during the study period was not available for all the studies included in this review.

Understanding the seasonality of an infectious disease and the driving factors are of utmost importance for planning prevention and control strategies [21,35]. Recently, several epidemiological models of CDI have been constructed to inform control strategies for this disease of increasing incidence and severity [36–39]. However, none has yet incorporated the effects of seasonality and this will be difficult to achieve without better understanding of the underlying mechanisms. The current review provided evidence of a similar CDI seasonal pattern across hemispheres which differs from the seasonality that was previously proposed. Further studies are required to identify exposure to medications and environmental factors associated with the observed seasonality.

## Supporting Information

S1 PRISMA Checklist (DOC)

S1 Table Excluded studies. (DOCX)

S2 Table Study quality assessment. (DOCX)

S1 Text Search Strategy. (DOCX)
