# Supplementary material for: Clostridium difficile Infection Seasonality: Patterns across Hemispheres and Continents – A Systematic Review
Source: PLoS One. 2015 Mar 16;10(3):e0120730. doi: 10.1371/journal.pone.0120730 (PMC4361656; doi:10.1371/journal.pone.0120730)
Supplement: S1 Table — (DOCX) [file pone.0120730.s002.docx]

**S1 Table.-** Characteristics of excluded studies

| Author, year | Reason for exclusion |
| --- | --- |
| Bignardi and Askew, 2000 ‡ | Reported the number of cases per month without the number of stool samples/patients tested |
| Cooper *et al.*, 2011 | Hydrogen peroxide vapour intervention was implemented to reduce the incidence of *C. difficile* |
| Denno *et al.*, 2005 § | Reported the number of cases per month without the number of stool samples/patients tested |
| Elumogo *et al.*, 2009 ‡ | Reported the number of cases per month without the number of stool samples/patients tested |
| Fekety *et al.*, 1997 § | Reported the number of cases per season without the number of stool samples/patients tested |
| Fellmeth *et al.*, 2010 ‡ | Reported the number of cases per season without the number of stool samples/patients tested |
| Feuerstadt *et al.*, 2013 ‡ | Reported the number of cases per season without the number of stool samples/patients tested |
| Garcia *et al.*, 2007 | Only 9 months follow-up |
| Gardilcic *et al.*, 2000 | Only 4 months follow-up |
| Gulacsi *et al.*, 2013 | In Hungarian |
| Hall *et al.*, 2012 | Measured mortality rates of *C. difficile* |
| Kim *et al.*, 1989 ¶ | Reported the number of cases per month without the number of stool samples/patients tested |
| Kyne *et al.*, 1998 | Only 7 months follow-up |
| Larang *et al.*, 2011 ¶ | Unable to extract data. Reported “A bimodal seasonal distribution of positive tests was noted with peaks in March and November”. |
| Marco-Martinez *et al.*, 2014 § | Reported the number of cases per season without the number of stool samples/patients tested |
| Pearson *et al.*, 2009 § | Reported the number of cases per season without the number of stool samples/patients tested |
| Polgreen *et al.*, 2010 ‡ | Reported the number of cases per month without the number of stool samples/patients tested |
| Polgreen *et al.*, 2011 ‡ | Reported the number of cases per month without the number of stool samples/patients tested |
| Riley *et al.*, 1994 § | Unable to extract data. Reported “A statistically significant seasonal variation in the isolation rate for *C. difficile* could not be demonstrated”. |
| Souza Dias *et al.*, 2010 § | Reported the number of cases per month without the number of stool samples/patients tested |
| van Kleef *et al.*, 2014 § | Reported the number of cases per month without the number of stool samples/patients tested |
| ‡ Corresponding author was contacted via email for further information regarding the number positive stool specimens for *C. difficile* and/or the total number of stool specimens/patients tested per months/seasons, no response was received.  § Corresponding author replied, but was unable to provide the information before the manuscript was submitted for review.  ¶ Unable to contact the corresponding author. | |
