# Supplementary material for: Clostridium difficile Infection Seasonality: Patterns across Hemispheres and Continents – A Systematic Review
Source: PLoS One. 2015 Mar 16;10(3):e0120730. doi: 10.1371/journal.pone.0120730 (PMC4361656; doi:10.1371/journal.pone.0120730)
Supplement: S2 Table — (DOCX) [file pone.0120730.s003.docx]

**S2 Table.-** Study quality assessment using the Newcastle-Ottawa Scale

|  | Representative of the exposed cohort | Selection of the non exposed cohort | Ascertainment of exposure | Demonstration that outcome of interest was not present at start of study | Comparability of cohorts on the basis of the design or analysis | Assessment of outcome | Was follow-up long enough for outcomes to occur | Adequacy of follow-up of cohorts | Total |
| --- | --- | --- | --- | --- | --- | --- | --- | --- | --- |
| Archibald *et al*., 2004 [[4](#_ENREF_4)] | 0 | NA | NA | 0 | NA | 1 | 1 | 1 | 3/5 |
| Brown *et al*., 2013 [[11](#_ENREF_11)] | 1 | 1 | 1 | 0 | 2 | 1 | 1 | 1 | 8/9 |
| Burckhardt *et al*., 2008 [40] | 1 | NA | NA | 0 | NA | 1 | 1 | 1 | 4/5 |
| Camacho-Ortiz *et al*., 2009 [[4](#_ENREF_40)1] | 1 | NA | NA | 0 | NA | 1 | 1 | 1 | 4/5 |
| Damani *et al*., 2011 [4[2](#_ENREF_41)] | 0 | NA | NA | 0 | NA | 1 | 1 | 1 | 3/5 |
| Deorari *et al*., 1999 [[34](#_ENREF_33)] | 1 | 1 | 1 | 1 | 2 | 1 | 1 | 1 | 9/9 |
| Dubberke *et al*., 2009 [[43](#_ENREF_42)] | 1 | NA | NA | 1 | NA | 1 | 1 | 1 | 5/5 |
| Faires *et al.*, 2014 [[44](#_ENREF_43)] | 1 | NA | NA | 1 | NA | 1 | 1 | 1 | 5/5 |
| Furuya-Kanamori *et al*., 2014 [[14](#_ENREF_14)] | 1 | NA | NA | 0 | NA | 1 | 1 | 1 | 4/5 |
| Gilca *et al*., 2010 [[5](#_ENREF_5)] | 1 | NA | NA | 0 | NA | 1 | 1 | 1 | 4/5 |
| Gilca *et al*., 2012 [[12](#_ENREF_12)] | 1 | 1 | 1 | 1 | 1 | 1 | 1 | 1 | 8/9 |
| Jagai and Naumova, 2009 [[6](#_ENREF_6)] | 1 | NA | NA | 0 | NA | 1 | 1 | 1 | 4/5 |
| MacDonald *et al.*, 1993 [[45](#_ENREF_44)] | 1 | NA | NA | 1 | NA | 1 | 1 | 1 | 5/5 |
| McFarland *et al.*, 2007 [[4](#_ENREF_45)6] | 1 | 1 | 1 | 0 | 2 | 1 | 1 | 1 | 8/9 |
| Reil *et al*., 2012 [[4](#_ENREF_46)7] | 1 | NA | NA | 0 | NA | 1 | 1 | 1 | 4/5 |
| Reveles *et al.*, 2014 [[4](#_ENREF_47)8] | 1 | NA | NA | 0 | NA | 1 | 1 | 1 | 4/5 |
| Slimming *et al*., 2014 [[15](#_ENREF_15)] | 1 | NA | NA | 1 | NA | 1 | 1 | 1 | 5/5 |
| Sonnenberg, 2009 [[4](#_ENREF_48)9] | 1 | NA | NA | 0 | NA | 1 | 1 | 1 | 4/5 |
| von Muller *et al*., 2011 [50] | 1 | NA | NA | 0 | NA | 1 | 1 | 1 | 4/5 |
| Wong-McClure *et al.*, 2012 [30] | 1 | NA | NA | 0 | NA | 1 | 1 | 1 | 4/5 |
| *NA:* Not applicable if it was a surveillance study that described the seasonal pattern of CDI | | | | | | | | | |
