# Supplementary material for: Clostridium difficile Infection Seasonality: Patterns across Hemispheres and Continents – A Systematic Review
Source: PLoS One. 2015 Mar 16;10(3):e0120730. doi: 10.1371/journal.pone.0120730 (PMC4361656; doi:10.1371/journal.pone.0120730)
Supplement: S1 Text — (DOCX) [file pone.0120730.s004.docx]

**S1.A.-** Search strategies

**PubMed**

("Clostridium"[Mesh] OR Clostridium))

AND

((Difficile)

AND

("Seasons"[Mesh] OR Season OR Seasons OR Seasonal)

**Embase**

'season'/exp OR season OR seasons OR seasonal

AND

'clostridium'/exp OR clostridium

AND

difficile

**LILACS Virtual Health Library**

Clostridium [Words]

AND

Difficile [Words]

**S1.B.-** Targeted search strategy for Southern hemisphere studies

**PubMed**

(((("Clostridium"[Mesh] OR Clostridium))

AND

Difficile

AND

((("Africa"[Mesh]) OR "Australia"[Mesh]) OR "South America"[Mesh] OR Africa OR Australia OR “South America” OR “Southern Hemisphere”)))
